# Eating Together but Often Feeling Lonely: Residents' Mealtime Experiences in a Nursing Home

**DOI:** 10.1111/jocn.17561

**Published:** 2024-11-20

**Authors:** Magdalena Nielsen, Carina Werkander Harstäde, Anna Sandgren

**Affiliations:** ^1^ Center for Collaborative Palliative Care, Department of Health and Caring Sciences Linnaeus University Växjö Sweden; ^2^ Department of Health and Caring Sciences Linnaeus University Växjö Sweden

## Abstract

**Aim:**

To explore residents' experiences of the mealtime environment in nursing home.

**Design:**

An exploratory qualitative design was employed to gain in‐depth insights.

**Methods:**

Twenty semi‐structured interviews were conducted with residents at a nursing home. Data were analysed using thematic analysis as outlined by Braun and Clarke. The consolidated criteria for Reporting Qualitative research checklist were used to support the research process.

**Results:**

Four main themes emerged from the analysis: (1) The significance of food, emphasising the centrality of food quality and variety in residents' mealtime experiences. (2) Security through routines, illustrating how established mealtime routines provide comfort and predictability. (3) Variability in staff influence, reflecting residents' perceptions of staff competence and their impact on the dining experience. (4) Limited social interactions, highlighting the varied social dynamics and their effects on residents' sense of community and isolation.

**Conclusion:**

The study underscores the critical importance of food quality, staff compliance and consistent routines in enhancing mealtime experiences in nursing homes. Additionally, it reveals that the ability to choose social interactions plays a significant role in residents' satisfaction and social well‐being.

**Implication for Patient Care:**

This study provides valuable insights for improving mealtime experiences in nursing homes, suggesting that person‐centred care and resident involvement in meal planning can enhance satisfaction and nutritional intake.

**Impact:**

The findings offer practical guidance for healthcare management, emphasising the need to prioritise and personalise mealtime environments to better meet residents' needs and preferences.

**Summary**

- The study provides insights into how nursing home residents, both with and without cognitive impairments, experience the mealtime environment and its impact on them.
- The findings of this study support the need for person‐centred care to optimise the nursing home mealtime environment.

## 1 Introduction

As the global population ages, the care of older persons has become a prominent societal discussion (European Union [EU] 2020; World Health Organisation [WHO] 2023). Long‐term care facilities, such as nursing homes, play a crucial role in maintaining older persons' well‐being. Long‐term care aims to ensure the well‐being and dignity of its residents (WHO 2021), which includes creating well‐functioning mealtime environments, and well‐being in the mealtime situation (Jung et al. 2021; Liu et al. 2020).

In Sweden, municipalities are required by law to facilitate nursing homes (Social Services Act 2001) which is an accommodation that needs an application to be granted by the Swedish social service. To move into such facility, the applicant must be over 65 years old and have a need for assistance to manage daily activities (Swedish Association of Local Authorities and Regions 2024). The highest prevalence of moving into a nursing home in Sweden are amongst persons over the age of 80 (The National Board of Health and Welfare 2023).

## 2 Background

Older adults often shift to long‐term care facilities, including nursing homes, because of loss of functional ability, cognitive impairment or the need for complex care resulting from conditions such as frailty (EU 2020). These issues necessitate assistance with daily activities, including mealtimes (Wu et al. 2023), which are significant for overall well‐being. Mealtimes in a nursing home typically occur in a common dining room where residents have individual preferences on how and what to eat, and varying needs for assistance (Jung et al. 2021; Liu et al. 2020). This complexity is likely to contribute to the challenge of optimising mealtime experiences. Moreover, mealtime in a nursing home also serves as a workplace, adding further complexity due to the involvement of staff and management (Gustafsson et al. 2006; Heikkilä et al. 2022).

The mealtimes can function as a structure during the day and holds a social meaning that can enhance the well‐being of the mealtime participants (Keller et al. 2024; Watkins, Goodwin, Abbott, Hall, et al. 2017). However, poorly functioning mealtimes in nursing homes can be a contributing factor to become malnourished which can result in poor health and reduced quality of life (Liu, Perkhounkova, and Hein 2022; Naseer, Forssell, and Fagerström 2016; Volkert et al. 2015).

Many nursing home residents have cognitive impairments, primarily due to dementia, which further adds to the complexity (Centers for Medicare and Medicaid Services 2016; Pakai et al. 2021; Takada et al. 2017; Watkins, Goodwin, Abbott, Hall, et al. 2017). Dementia often affects independence during mealtimes, resulting in both cognitive and physical challenges (Pakai et al. 2021; Takada et al. 2017). The mealtime needs and perceptions of residents with cognitive impairments differ from those without such impairments, influencing the type of care required. Dependency during mealtimes is linked to malnutrition, with a lack of independence being common amongst individuals with cognitive impairment (Pakai et al. 2021; Takada et al. 2017; Watkins, Goodwin, Abbott, Hall, et al. 2017).

A well‐functioning mealtime environment in a nursing home is associated with higher food and nutrient intake (Liu, Jao, and Williams 2017; Morrison‐Koechl et al. 2021). Positive social interactions during mealtimes can enhance residents' well‐being beyond the nutritional benefits. This contributes to the well‐being that a sufficient intake of nutrients brings (Keller et al. 2024; Watkins, Goodwin, Abbott, Hall, et al. 2017).

The mealtime environment can be explained using the theoretical model, the Five Aspect Meal Model [FAMM] (Gustafsson et al. 2006). FAMM includes both physical and psychosocial aspects of the mealtime environment: the room, meeting, product, management control system and atmosphere. Physical aspects include the room and product such as furnishing and food, whereas the psychosocial aspects encompass the meeting, management control system and atmosphere. All five aspects affect each other and must function well for the mealtime participants to ensure a positive mealtime experience, which refers to a person's experience of the mealtime environment (Gustafsson et al. 2006). Therefore, the need to optimise the mealtime environment is important as the experience influences both general well‐being as well as nutrient intake (Keller et al. 2024; Liu, Jao, and Williams 2017; Morrison‐Koechl et al. 2021; Watkins, Goodwin, Abbott, Hall, et al. 2017).

Research on mealtime environments and experiences of the mealtime in nursing homes is becoming increasingly common, although further research is necessary to plan effective interventions and implement new strategies. For example, comprehensive studies exploring various aspects of residents' mealtime experiences are essential (Jonsson et al. 2021; Pakai et al. 2021; Watkins, Goodwin, Abbott, Hall, et al. 2017; Watkins, Goodwin, Abbott, Backhouse, et al. 2017). Therefore, this study aims to explore residents' experiences of the mealtime environment in a nursing home.

## 3 Method

### 3.1 Study Design

This study employed an explorative qualitative design to gain deeper insights into residents' experiences of the mealtime environment in a nursing home. Thematic analysis according to Braun and Clarke (2006) was used to capture experiences, meanings and the reality of participants involved in the study.

### 3.2 Study Setting and Recruitment

The study was conducted in a nursing home with municipal management in southern Sweden, comprising 85 resident apartments divided into 9 units, 6 of which specialised in dementia care. Each unit had its own dining room, and meals were prepared centrally in a municipal kitchen, located separate from the nursing home, and delivered to the nursing home in portable heating cabinets. Breakfast was prepared in the units' kitchens.

Initially, the researcher contacted the staff in the units to identify potential interviewees amongst the residents. Staff members then approached these residents to inquire if they were willing to meet with the researcher. The staff verbally explained the purpose of the meeting and the aim of the study. All residents who were approached agreed to an initial meeting with the researcher. During this meeting, the researcher provided information about the study and asked if the resident would be willing to participate in an interview. Subsequently, all residents who were asked agreed to participate. Written consents were given from those who could sign, verbal consent was given by those who, due to physical inability, could not sign. Interview times and locations were then arranged based on residents' preferences, with interviews often conducted soon after or in conjunction with the initial meeting. The residents decided where and when the interview would occur.

### 3.3 Inclusion and Exclusion Criteria

Purposeful sampling ensured adherence to the inclusion criteria, which required residents to live in the nursing home and understand the study information provided to facilitate their informed consent for participation in the study. The second criterion was crucial due to the high prevalence of dementia among residents, ensuring ethical considerations were met. Importantly, residents with cognitive impairments were not excluded, reflecting the demographic composition of the nursing home.

### 3.4 Data Collection

Twenty residents were approached to participate in the study, and all agreed to participate. They had been residing in the nursing home for 2 months to 5 years and included 13 women and 7 men, aged between 78 and 96 years. Ten participants lived in units specialised in dementia care (6 women and 4 men), while ten lived in non‐specialised units (7 women and 3 men). The interviews were conducted over a period of 8 months.

Before the interviews, the residents were provided with the option to have a next of kin or a staff member present during the interviews for support, but all declined. The residents chose the interview location. All interviews except three were conducted in the residents' apartments at the nursing home. Three interviews occurred in alternative locations: two in common rooms with closed doors and one in a secluded area. The first author conducted all interviews, which lasted between 15 and 40 min. All interviews, except one, were recorded and fully transcribed by the first author. One resident declined to record, and therefore, notes were taken.

The interviews were semi‐structured and began with a broad question about their mealtime or the mealtime environment experiences. Follow‐up questions were then asked, depending on the answers and the person being interviewed. FAMM provided a framework to ensure a comprehensive exploration of the mealtime environment.

### 3.5 Data Analysis

The data were analysed using thematic analysis, according to Braun and Clarke (2006). This method was considered appropriate as the study aimed to identify, analyse and report patterns in residents' mealtime environment experiences in a nursing home. The data were read thoroughly and repeatedly to ensure familiarity with the content. Once familiar, the first author initiated the next step of the analysis, extracting and coding data segments. These codes were then compared with coding generated by the second author to ensure inter‐rater reliability. Continuous discussions amongst all authors were then held throughout the analysis to gain an enhanced the understanding of the data. During these discussions, the coding and grouping was changed and rearranged when going back and forth between the data and the codes and subthemes were detected. Themes were subsequently identified, reviewed and refined in relation to the data. Examples of the analysis process are presented in Table 1. In the final step, the data were compared against the themes to confirm their validity and connection to the codes (Braun and Clarke 2006).

**TABLE 1:** Examples of the analysis process.

Data extract	Code	Subtheme	Theme
Resident: It is almost the same food that was for lunch. It is leftovers, almost the same food that was for lunch	Negative experiences of the food	Quality of food is of importance	The significance of food
Interviewer: Do you have fixed places? Resident: Yes we have. No one has decided on it, but you sit in a certain place. We are people of habit	Designated places at dining table have developed	Routines gives calm	Security through routines
Resident: No, oh no, they know that. Yes, yes. They know us who is here and know how we want it. So, I get that, and as I said, if I want more or different you can just tell them. So that is good	They have learnt how we prefer it	Staffs' knowledge of personal preferences provides simplicity	Variability in staff influence
Interviewer: Is there no one to talk to? Resident: No, I do not think I have that. But then there is maybe someone who is clearer in the head and a bit more to talk to, but then they sit a bit away and then it does not work	Difficulties to find someone to talk to	Lack of social meetings at the mealtime	Limited social interactions

### 3.6 Ethical Considerations

The nursing home was part of a collaborative project between the municipality and the research team, and the study was approved by the management. The Swedish Ethical Review Authority also approved the study (dnr 2019‐05477). All participants received verbal and written information, and all provided informed consent to participate in the study, following the Declaration of Helsinki for Human Research (World Medical Association [WMA] 2013).

### 3.7 Rigour and Reflexivity

The study's quality was ensured by adhering to Braun and Clarke's (2006) 15‐point checklist for evaluating thematic studies. Furthermore, the authors continuously reflected on their own reflexivity throughout the study. The first author had previous experience with mealtime environments in nursing homes, both from clinical work as a nurse and from prior research in the context. The first author, who performed all interviews, also had extensive experience communicating with individuals diagnosed with dementia. These experiences were subjects for discussion and reflection amongst the authors during the process of data collection and analysis to ensure being true to the data (Braun and Clarke 2023).

By using the Consolidated criteria for Reporting Qualitative research checklist (COREQ) Appendix S1, trustworthiness was determined, as the COREQ checklist promotes quality and trustworthiness of qualitative research (Tong, Sainsbury, and Craig 2007).

## 4 Results

The residents' mealtime environment experiences at the nursing home are explained by four themes: the significance of food, security through routines, variability in staff influence and limited social interactions.

### 4.1 The Significance of Food

When asked about the mealtime and its environment, residents primarily focused on their experiences with the food. They expressed that food was the most critical aspect of mealtime. Overall, their experiences with the food were positive. The meals consisted of familiar dishes they had previously eaten or even cooked themselves, which created expectations about how the food should taste. Residents often compared the food at the nursing home with the food they had at home, not necessarily to critique but to note the differences. The most positive aspects highlighted were the taste and the variety of the dishes.

It is good, there is nothing wrong with it. It is food I know… varied. (13)

Residents' negative experiences with the food were most often related to the texture of the food. Raw vegetables were described as being sometimes too hard to chew because of residents' dental issues, and other components of the dish occasionally hardened because of being cooked somewhere else and then transported to the nursing home. These issues made the meal less enjoyable and sometimes led residents to leave their food unfinished.

Then I save them [the raw vegetables] for last, and then I sit like this and try to chew them. (8)

Breakfast and lunch were the most favoured meals, while supper was the least liked. Residents often described supper as leftovers from lunch, leading some to skip this meal. Additionally, supper was the meal that most residents chose to eat in their own apartments instead of the common dining room.

Here at least there are a lot of us who do not like supper or very rarely eat supper. (2)

I skip the evening meal… because it is the same food as lunch. (16).

### 4.2 Security Through Routines

This theme highlights the importance residents attach to routines and the sense of security and calmness routines bring to the mealtime experience. It also addresses the disturbances caused when these routines are disrupted or altered.

Residents emphasised the comfort of having designated places at the dining table. They did not attribute this arrangement solely to staff decisions and were often unsure sure how these places were assigned. Sometimes residents themselves developed these routines. While they may not have always liked their assigned seats, they appreciated the idea of having designated spots. No resident expressed any negative opinions about routines, in relation to the mealtime, being set without their involvement.

Residents described the common dining room as pleasant and accessible, although it often accommodated several walking aids and some wheelchairs simultaneously. Navigating to the dining table could be challenging due to the numerous aids, but residents considered these situations manageable and non‐disruptive. They often helped each other, ensuring these circumstances did not become significant obstacles.

Yes, sometimes it can be a little crowded, of course, it can be. But, we work it out. (18)

Residents reported that staff always served their food, either from a serving trolley or canteens on a kitchen counter. None of the residents served themselves, and they viewed this arrangement as the best option, citing various reasons. The primary reason was related to hygiene; residents believed that others in worse conditions might create a messy and less clean food area. Physical impairments also rendered self‐serving difficult, such as walking with a plate or using a wheelchair. Even those who could walk with their plate preferred being served.

How many are we in the department, 10–12? Everyone is old and it does not work. Almost all of them are worse than me, and it feels the cleanest, most appetising, and that there are no others there and touching [the food]. (8)

Residents expressed dissatisfaction with recent changes in the routine regarding food delivery times, leading to delays in meal service. This shift in routine had happened sometime during the last months. They found waiting for food and not knowing when they would eat challenging and frustrating. These delays sometimes caused meals to be too close together or too far apart, which displeased them. This issue was a primary concern for residents, often leading them to choose to eat the meals in their own apartments instead of the common dining room, where they could decide when to eat. This flexibility was perceived as positive and sometimes necessary, especially when they were not hungry when the food arrived at the nursing home.

### 4.3 Variability in Staff Influence

Residents' reflections on their experiences of the staff's involvement in the mealtime environment revealed that staff significantly impacted some aspects of mealtime while having minimal influence on others. Residents often mentioned staff impact in relation to the service provided. However, they did not attribute much importance to staff in the social context. Sometimes, residents viewed staff as fellow meal participants sitting to eat with them, while other times, they did not notice the staff's presence. When residents noticed staff sitting down to eat with them, they sometimes questioned the reasons, perhaps because they did not experience them sitting down, as being part of the mealtime together with the residents. They did not always perceive staff participation as a positive interaction but rather as staff taking a break and interacting amongst themselves, which could be loud and disruptive. This perceived lack of attention from staff towards residents was disturbing, although some residents found it pleasant when staff joined them at the dining room table.

[staff] eats and talk about theirs. Often, there is a lot of babbling, which is a bit disturbing. One time, I raised the alarm because we could not get their attention even if we were sitting in the same room. (17)

The residents discussed their experiences of how staff served the food, emphasising the importance of an optimal staff size—neither too few nor too many. When the number of staff was just right, residents experienced the situation as smooth and clear, bringing a sense of ease. Conversely, too many or too few staff created an unstructured and confusing environment.

Another important component for a comfortable and calm mealtime was the staff knowledge of residents' personal preferences. Residents appreciated when staff knew their food preferences and portion sizes, or required assistance. Residents further described how attentive staff made the mealtime easier and more enjoyable, reducing the need for residents to explain their needs. Conversely, when staff lacked knowledge of residents' preferences, mealtime became difficult. Worse still was the experience of staff not knowing how to perform their mealtime duties properly. Residents attributed these issues to inexperienced, uneducated or substitute staff. However, they did not blame the staff personally, describing them as sweet and hardworking and emphasised that staff were doing their best given the conditions provided by management.

### 4.4 Limited Social Interactions

Residents often described their mealtime company as merely the other residents, seldom referring to them as friends or people they enjoy socialising with. While they acknowledged helping each other and always having someone to talk to, more often they experienced a difference between themselves and others during mealtimes. This perceived difference was often characterised by distinctions in age and the level of help needed, making them feel incompatible with their dining companions. Some residents expressed feelings of loneliness, which could have been alleviated by having more compatible company.

And, it seems to me that most of them are pretty unwell. This far anyway. I do not think there is anyone who is as well as me. (6)

It is so damn hard there because there is no one you can talk to directly among the people who live here. I feel so damn isolated. (15)

Residents also recounted unpleasant incidents during mealtime due to the behaviour of other residents. These situations sometimes led to a habit where they avoided staying in the dining area longer than necessary and to steer clear of disturbances.

Then it might be a bit of a squabble, it is the old men who fuss the most, “giggles,” and things like that, and that is, people come here who swear and disturb, so you get to be part of quite a lot here you have to say, I try to stay away. (12)

Despite the lack of social connections during mealtimes, residents generally appeared content with the situation. They often viewed mealtime as an opportunity to eat and then preferred to return to their rooms for various reasons, such as poor hearing, introverted personalities or fatigue.

Yes, because I am like I say, I am not very talkative otherwise, but I think it is quite pleasant to be able to return to my own living space. (9)

Residents also exhibited a strong sense of acceptance. They discussed their experiences, both positive and negative, highlighting what they had and what they missed. They acknowledged that everything could not be as it once was and expressed acceptance of this reality and contentment with their current situation. They recognised that sharing meals with others meant their individual preferences could not always be met.

And then you have learned, you cannot hang on and get hung up on trifles, it does not work, then nothing will be good. Try and look over the edge instead and you will be fine. (3)

## 5 Discussion

This study demonstrates that residents' experiences of the mealtime environment vary significantly. The themes—significance of food, security through routines, variability in staff influence and limited social interactions—highlight common aspects that residents found important about the mealtime environment, yet their experiences of these aspects differed. These diverse experiences can probably be attributed to residents' different preferences, need for assistance and cognitive abilities, as described in various studies (Faraday et al. 2021; Jung et al. 2021; Liu et al. 2020). The varying experiences observed in this study are consistent with the need for person‐centred care in this context, as detailed in studies and guidelines (Liu, Jao, and Williams 2017; Liu, Perkhounkova, and Hein 2022; WHO 2021).

Using the FAMM as a foundation for defining and exploring the mealtime environment elicited a combination of residents' experiences related to both physical and psychosocial aspects. This comprehensive understanding is essential for grasping the complex situation that mealtime in nursing homes represents. The holistic perspective that the FAMM provides is needed for developing mealtime environments in nursing homes (Heikkilä et al. 2022).

Regarding the psychosocial aspect of the mealtime environment, the staff's knowledge, professionalism and compliance appeared to be more influential than social interactions between residents. Although residents focused heavily on the physical aspects of the mealtime environment, food remained the most important aspect, encompassing the type of food, its taste and texture, which facilitated easier consumption. The interviewed residents did not relate their food intake to their experiences of the psychosocial aspects, although studies often indicate that the social context influences food intake (Liu, Jao, and Williams 2017; Morrison‐Koechl et al. 2021). According to the residents' descriptions, they appeared to explain their food consumption based on their experience of the food. This result indicates that optimising the mealtime environment necessitates the inclusion of residents during menu planning, thereby highlighting the key role of the management control system in the mealtime experience (Gustafsson et al. 2006). Including residents in menu planning should be feasible, regardless of cognitive ability, although its commonality of implementation is unknown (Milte et al. 2017).

When residents discussed the social aspects of the mealtime environment, their diverse experiences became evident. They mostly focused on the presence of other residents during mealtimes rather than their own fulfilled preferences. Nevertheless, the experience differed, indicating that person‐centred arrangements at these mealtimes could be beneficial, potentially focusing on residents causing difficult situations (Davies et al. 2022; Liu, Perkhounkova, and Hein 2022). The most distinct social experience was the feeling of having no one to talk to during mealtime, which some residents expressed as acceptable. In relation to studies describing the social importance of mealtimes, this experience could be considered suboptimal (Keller et al. 2024; Watkins, Goodwin, Abbott, Hall, et al. 2017). The sense of acceptance concerning various aspects of the mealtime environment, even when not ideal, was present across all themes. This acceptance could be interpreted as a resignation of the situation, although that is not how the residents expressed it. Instead, the acceptance seemed to reflect an understanding that living in a nursing home entailed adjustments and compromises to enable the smooth functioning of the organisation. This understanding raises questions about whether such acknowledgement is acceptable regarding the social aspect of the mealtime environment. A study on barriers to social connections in nursing homes reveals that residents' preferences for social interactions can shift depending on their surroundings, leading to a loss of interest in participating socially due to a lack of compatible company (Abbott et al. 2018). Perhaps the acceptance of the lack of conversations during mealtimes experienced by residents also represents such a shift in preferences.

Studies indicate that eating in a positive social context increases energy intake (Liu, Jao, and Williams 2017; Morrison‐Koechl et al. 2021) and enhances well‐being regardless of nutrient intake (Keller et al. 2024; Watkins, Goodwin, Abbott, Hall, et al. 2017). However, whether this positive social context is solely due to socialising or also due to residents' ability to choose when and how much they want to socialise. Having options for where and with whom to eat is also highlighted as an important factor in another interview study with residents in care homes (Watkins, Goodwin, Abbott, Hall, et al. 2017). This raises questions about the causes of these effects. Various studies describe how independence and the opportunity to choose are important for the well‐being of residents in nursing homes (Davies et al. 2022; Faraday et al. 2021; Roos et al. 2022). Maybe the opportunity to select is as important for the well‐being as social interactions in the mealtime environment (Keller et al. 2024; Watkins, Goodwin, Abbott, Hall, et al. 2017). Moreover, the residents discussed the ease of mealtime when staff knew them well. This familiarity with staff may not only facilitate smooth routines but also contribute to a form of positive social context. This finding further strengthens the conclusion that person‐centred care is essential in this context to ensure residents' well‐being and adequate nutrient intake (Douglas et al. 2021; Liu, Jao, and Williams 2017; Liu, Perkhounkova, and Hein 2022; WHO 2021).

Previous research highlights the mealtimes function as an event that facilitates daily structure for residents in nursing homes (Keller et al. 2024; Watkins, Goodwin, Abbott, Hall, et al. 2017). This function is evident in this study as well. The residents' strong opinions about the change in routines when the food arrived at the nursing home indicate that they plan their day largely based on meal times. This also underscores the importance of established routines. Residents mostly questioned the uncertainty about when the food would arrive rather than the change in routines (Keller et al. 2024; Watkins, Goodwin, Abbott, Hall, et al. 2017). This discussion returns to the earlier point about acceptance, where residents seem to feel that compromises are necessary, but it also emphasises the need to include residents in decisions affecting them. The question can be raised if management knows the effect this has on the residents. The inclusion of residents in decision‐making processes could prevent annoyances and improve their experience (Berge et al. 2022; Watkins, Goodwin, Abbott, Hall, et al. 2017).

No clear pattern was found to suggest that the experiences differed between residents living in dementia units and those living in non‐specialised units. The experiences varied between individuals. What sometimes differed between residents living in dementia units compared to those living in non‐specialised units was the lower level of detail in their experience descriptions. Nevertheless, the descriptions contained valuable information, contributing to the study's results. Including residents with a dementia diagnosis can be viewed as a strength of this study. As described in other studies, inclusion allows care to be developed with them, rather than just for them (Bartlett, Milne, and Croucher 2019; Frank et al. 2021). Without their inclusion, this study would not fully represent nursing home residents.

## 6 Methodological Considerations

Using the five aspects of FAMM as the interview structure helped ensure the inclusion of all elements important to the resident's mealtime experiences (Gustafsson et al. 2006).

Given the cognitive impairment amongst many interviewed residents, the interviews generally resembled conversations rather than structured interviews. Direct questions about a detailed subject were sometime difficult for residents to answer, frequently eliciting responses such as “Now you made it difficult for me” or “I do not remember.” Conversely, when the conversation about the mealtime environment was more informal and without direct questions, this concern did not arise. Additionally, when questions were too subjective, many residents became puzzled and either diverted to a different topic of their choice or did not respond at all. This required the researcher to be sensitive to the resident's needs and be adaptable in their approach. Despite these challenges, the inclusion of residents living in dementia units and their contributions to the results is considered a strength of the study. Research indicates that contributing to studies can be empowering for individuals with dementia, and their input is crucial for developing effective care practices (Bartlett, Milne, and Croucher 2019; Frank et al. 2021).

A limitation of the study is that it was conducted at a single site. The residents in the nursing home are quite homogeneous, with almost none of the older adults having an immigrant background and only one of the interviewed residents being an immigrant. This homogeneity is not representative of the diversity often found in Swedish nursing homes, which may limit the generalisability of the findings (The National Board of Health and Welfare 2023).

## 7 Conclusion

This study provided insights into how nursing home residents, both with and without cognitive impairments, experience the mealtime environment and its impact on them. Employing the theoretical FAMM framework, the study demonstrated that residents prioritised the quality of the food above all else. Regarding the psychosocial aspect of the mealtime environment, the staff's knowledge, professionalism and compliance were deemed more important than social interactions amongst residents. The ability to make personal choices regarding interactions with other residents was also significant. The residents' or others' conditions also influenced the opportunities and willingness to socialise. The findings of this study support the need for person‐centred care, with conversations being a primary tool in optimising the nursing home mealtime environment.

Further studies are needed to gain deeper knowledge of the mealtime environment. As the residents are not the only participants in the mealtime environment, exploring the staff's experience is also essential. Additionally, management at various levels has a significant influence on the mealtime environment, warranting further study to enhance understanding and improvement. Nursing home administrators, healthcare providers, policymakers and researchers are encouraged to collaborate in creating environments where residents feel heard and valued. Committing to further exploration and application of these findings is essential to transform mealtime experiences and enhance the overall quality of life for residents. Ensuring mealtimes are nourishing for both body and soul will drive continuous improvement in the care of the aging population.

## Author Contributions

Design: M.N., C.W.H., A.S.; data collection: M.N.; data analysis: M.N., C.W.H., A.S.; drafting of the manuscript: M.N., C.W.H., A.S.; and involvement in critical revision of the manuscript for important intellectual content: M.N., C.W.H., A.S.

## Consent

The authors have nothing to report.

## Conflicts of Interest

The authors declare no conflicts of interest.

## Supporting information


Appendix S1.

## Data Availability

The data that support the findings of this study are available from the corresponding author upon reasonable request.
