# Supplementary material for: Eating Together but Often Feeling Lonely: Residents' Mealtime Experiences in a Nursing Home
Source: J Clin Nurs. 2024 Nov 20;34(1):259–67. doi: 10.1111/jocn.17561 (PMC11655421; doi:10.1111/jocn.17561)
Supplement: Supplementary file 1 — Appendix S1. [file JOCN-34-259-s001.docx]

| **Domain 1: Research team and reflexivity** |  |
| --- | --- |
| **Personal Characteristics** |  |
| 1. Interviewer/facilitator Which author/s conducted the interview or focus group? | The first author conducted all interviews. Page 6. |
| 2. Credentials What were the researcher’s credentials? E.g. PhD, MD | MN: RN, PhD-student.  CWH: RN, PhD, Associate Professor.  AS: RN, PhD, Associate Professor. |
| 3. Occupation What was their occupation at the time of the study? | MN: PhD-student  CWH: Associate Professor in Caring Sciences  AS: Associate Professor in Caring Sciences |
| 4. Gender Was the researcher male or female? | Female |
| 5. Experience and training. What experience or training did the researcher have? Relationship with participants | Well experienced in holding conversations with persons diagnosed with dementia. Experience in conducting interviews related to qualitative studies. No prior relation to any of the participants. |
| 6. Relationship established. Was a relationship established prior to study commencement? | The author that conducted the interviews had visited the units multiple times before. There were no personal relationships between researcher and participants. |
| 7. Participant knowledge of the interviewer What did the participants know about the researcher? e.g. personal goals, reasons for doing the research | Written and oral information about the study were given. Page 5. |
| 8. Interviewer characteristics What characteristics were reported about the interviewer/facilitator? e.g. Bias, assumptions, reasons and interests in the research topic | The interviewers’ characteristics were discussed and reported in the manuscript according to Braun and Clarke’s (2006) 15-point checklist. Page 8. |
|  |  |
| **Domain 2: study design** |  |
| **Theoretical framework** |  |
| 9. Methodological orientation and Theory. What methodological orientation was stated to underpin the study? e.g. grounded theory, discourse analysis, ethnography, phenomenology, content analysis | Thematic analysis. Page 6. |
| **Participant selection** |  |
| 10. Sampling How were participants selected? e.g. purposive, convenience, consecutive, snowball | Purposeful sampling. Page 5. |
| 11. Method of approach How were participants approached? e.g. face-to-face, telephone, mail, email | Face-to-face. Page 6. |
| 12. Sample size How many participants were in the study? | 20 participants. Page 6. |
| 13. Non-participation How many people refused to participate or dropped out? Reasons? | No one declined to participate. Page 5. |
| **Setting** |  |
| 14. Setting of data collection Where was the data collected? e.g. home, clinic, workplace | In the participants’ apartments in the nursing home or privately in a room in the units. Page 6. |
| 15. Presence of non-participants Was anyone else present besides the participants and researchers? | No. |
| 16. Description of sample What are the important characteristics of the sample? e.g. demographic data, date | Yes. Page 5-6. |
| 17. Interview guide Were questions, prompts, guides provided by the authors? Was it pilot tested? | Semi-structured interviews were conducted with the aspects of FAMM as main questions. Page 6.  Not pilot tested. |
| 18. Repeat interviews Were repeat interviews carried out? If yes, how many? | No. |
| 19. Audio/visual recording Did the research use audio or visual recording to collect the data? | Audio recording was used after permission from the participants. One participant declined and more extensive fieldnotes were taken instead. Page 6. |
| 20. Field notes Were field notes made during and/or after the interview or focus group? | During and after. |
| 21. Duration What was the duration of the interviews or focus group? | The interview lasted between 15 to 40 minutes. Page 6. |
| 22. Data saturation Was data saturation discussed? | Theoretical saturation was discussed amongst researchers. All researchers were in agreement that theoretical saturation had been achieved. |
| 23. Transcripts returned Were transcripts returned to participants for comment and/or correction? | No. |
|  |  |
| **Domain 3: analysis and findings** |  |
| **Data analysis** |  |
| 24. Number of data coders How many data coders coded the data? | Three. |
| 25. Description of the coding tree. Did authors provide a description of the coding tree? | Description of the analysis and example of the analysing process are provided. Page 6, 7. |
| 26. Derivation of themes. Were themes identified in advance or derived from the data? | Derived from the data. |
| 27. Software What software, if applicable, was used to manage the data? | N/A |
| 28. Participant checking. Did participants provide feedback on the findings? | No. |
| **Reporting** |  |
| 29. Quotations presented Were participant quotations presented to illustrate the themes / findings? Was each  quotation identified? e.g. participant number | Yes. Each quotation is identified with a participant number. |
| 30. Data and findings consistent Was there consistency between the data presented and the findings | Yes. Page 8-12. |
| 31. Clarity of major themes Were major themes clearly presented in the findings? | Yes. Page 8-12. |
| 32. Clarity of minor themes Is there a description of diverse cases or discussion of minor themes? | No. |
